# Delivering on the promise of precision cancer medicine

**DOI:** 10.1186/s13073-016-0373-1

**Published:** 2016-10-25

**Authors:** Michael F. Berger, Eliezer M. Van Allen

**Affiliations:** 1 Department of Pathology, Memorial Sloan Kettering Cancer Center, New York, NY 10065 USA; 2 Human Oncology and Pathogenesis Program, Memorial Sloan Kettering Cancer Center, New York, NY 10065 USA; 3 Department of Medical Oncology, Dana-Farber Cancer Institute, Boston, MA 02215 USA; 4 Cancer Program, Broad Institute of MIT and Harvard, Cambridge, MA 02142 USA

The emerging paradigm of precision cancer medicine, in which the use of molecular data at the point of care directly impacts patient treatment and clinical decision-making, has already had a substantial, direct clinical impact on many aspects of medical oncology. Advances in precision cancer medicine include well-established molecularly targeted therapies for patients with *BRAF*-mutant metastatic melanoma [1], those with *EGFR*-mutant [2] or *ALK*-mutant [3] non-small-cell lung cancer, and those with *BCR–ABL-*translocation-positive chronic myelogenous leukemia [4]. In addition, the introduction of large-scale genomic technologies at the point of care [5–7] has catalyzed discoveries in translational oncology and is driving new research that aims to dissect selective responses to targeted, immune-driven therapies and to chemotherapies, as well as identifying novel targets for which therapies are now in development. Indeed, propelled by the ability to generate increasingly complex molecular data directly from patient tumor and germ-line samples, the cycle of translating discoveries into clinical practice may be accelerating.

However, precision cancer medicine remains a promise rather than a solid and robust reality in many areas of clinical oncology. Molecular profiling of tumors is not yet broadly applied in all types of cancer. Furthermore, although the molecular landscapes of many cancers have been revealed using precision oncology approaches, many of the alterations observed in patients remain undruggable, and viable targets are incompletely characterized. In addition, a considerable amount of diversity exists regarding the types of molecular tests being offered clinically [8]; combined with the knowledge gap regarding the interpretation of genomic test results within the field of clinical oncology, this diversity has fostered confusion among physicians about the meaning and the clinical utility of genomic data at the point of care [9]. Perhaps most critically, prospective trials of molecular profiling across cancers in an unselected manner are not yet mature. In aggregate, these issues have limited the practical applicability of precision cancer medicine, but they also define the areas in which investment is needed to make it a reality.

In this special issue of *Genome Medicine*, original research and reviews from leaders in the field tackle the current challenges in precision oncology. This special issue details technological and analytical advances in tumor characterization, which have provided novel insights into the cancer genome and epigenome. In addition, reports herein explore the integration of molecular profiling into clinical oncology to enable careful assessment of the extent to which prospective clinical sequencing is influencing treatment decisions and improving outcomes, for both adult and pediatric cancers. Furthermore, as new targets are discovered and novel drug resistance mechanisms emerge, combination therapies may prove to be advantageous, and promising therapeutic strategies in immuno-oncology will require new paradigms beyond the assignment of molecularly targeted therapies that are based on a single target lesion [10]. Improved methods to interpret genomic variants and integrate genomic data into the electronic health record [11] are essential for extending effective treatment options to patients who are most likely to benefit. In addressing all of these topics, this special issue provides a wide view of the current state of the field and highlights the breadth of translational and clinical advances enabled by the application of genomic approaches to the characterization, diagnosis, and treatment of cancer.

To capitalize on these advances and ensure maximal benefit to patients, we advocate expanded access to genomic testing for patients with all types of cancer. Currently, reimbursement for tumor molecular profiling is mostly limited to a small number of tumor types such as lung cancer and melanoma, for which standard-of-care therapies are administered on the basis of molecular aberrations. However, it is now well established that individual targetable alterations may occur across a wide range of histologically defined tumor types and that the same drug may elicit responses across different cancers. Furthermore, broadening the application of molecular profiling will be necessary to establish the evidence base for genomically guided therapy for patients with rare cancers and for patients with common cancers that have rare mutations. Innovative clinical trials such as “basket studies” [12], which enroll patients on the basis of a specific genomic alteration that is shared across many histologies, are essential for increasing access to promising therapies and for evaluating the degree to which drug response is determined by disease context. Large academic medical centers have primarily funded large-scale clinical sequencing through philanthropic and research funds. This trend is unsustainable and it is incumbent upon all stakeholders to ensure that tumor genomic profiling is made available to all who might benefit from it.

With expanded access to genomic profiling, obtaining the necessary data regarding treatment outcomes and carrying out prospective randomized controlled trials may become more feasible at the scale necessary to determine the clinical impact of this approach across cancer types, given the clinically heterogeneous patient populations that would receive such testing. In addition, this approach will highlight areas in which current methodology is insufficient and in which other profiling approaches, such as transcriptome or epigenetic profiling, may provide further clinical impact. Towards that end, studies in prostate [13] and pediatric [14] cancers have already indicated the benefit of adding transcriptome-level data to genome-level data to identify actionable targets. Such an approach may also inform multi-omic features that are associated with response and resistance to emerging immuno-oncology treatment paradigms, which may foster new methods for patient stratification and highlight resistance mechanisms that, in turn, may identify novel combination strategies.

Finally, a complete assessment of the utility of genomic profiling in clinical oncology will require genomic and clinical data to be shared across institutions. Data-sharing is a central tenet of the Cancer Moonshot initiative [15] advocated by Vice President Biden, and the National Cancer Institute recently launched the Genomic Data Commons to centralize and standardize cancer genomic data [16]. Given the rarity of many putative targetable mutations in cancer, data-sharing among institutions and clinical laboratories engaged in tumor sequencing is necessary to prioritize novel biomarkers that may inform future treatment decisions. Furthermore, data-sharing driven by cancer patients themselves may complement these approaches through patients’ right of access to medical records. Integrating data generated by multiple platforms and modalities may enable the identification of novel genetic targets that would go undetected if the data remain in silos. Ultimately, these steps are necessary to build on the efforts described in this special issue and to bring us closer to truly delivering on the promise of precision medicine in cancer.
